# Supplementary material for: Prolonged Sleep Deprivation Induces a Reprogramming of Circadian Rhythmicity with the Hepatic Metabolic Transcriptomic Profile
Source: Biology (Basel). 2024 Jul 17;13(7):532. doi: 10.3390/biology13070532 (PMC11274269; doi:10.3390/biology13070532)
Supplement: Supplementary file 1 [file biology-13-00532-s001.zip › Figure S1 and Table S1.pdf]

Supplemental information

Figure S1

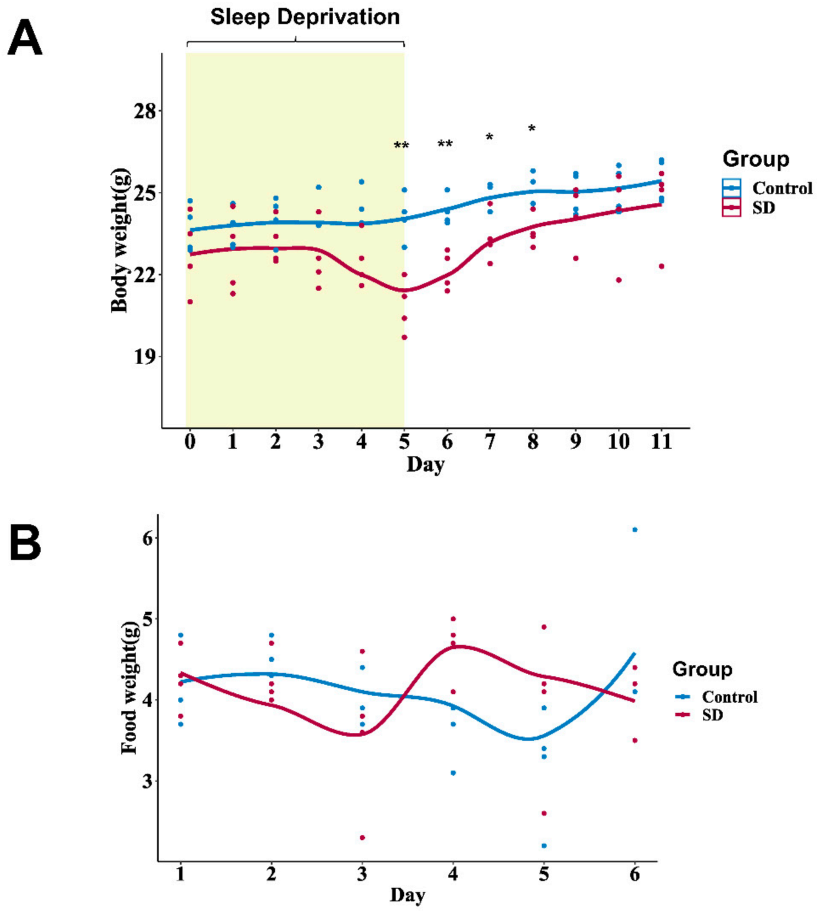

Figure S1. (A)Body weight change curves (n = 4 mice per group). (B) Plot of daily food intake after 5 days of sleep deprivation (n = 4 mice per group). Statistical significance represents the comparison between Control and SD group as per unpaired Student's t-test. **p <0.05, **p < 0.01, ***p < 0.001*, see File S4.

Table S1. Real-Time PCR primer sequences

Gene		Primer sequences (5′-3′)
Bmal1	Forward	CACCGTGCTAAGGATGGCTG
	Reverse	CTGCTGCCCTGAGAATTAGG
Dbp	Forward	CGTGGAGGTGCTAATGACCTTT
	Reverse	CATGGCCTGGAATGCTTGA
Rorc	Forward	TCCACTACGGGGTTATCACCT
	Reverse	AGTAGGCCACATTACACTGCT
Clock	Forward	TCACCACGTTCACTCAGGACA
	Reverse	AAGGATTCCCATGGAGCAA
Cry2	Forward	ACAACCGAAGCGGAGATAAG
	Reverse	TCCCAAGGCTGTTCAAGG
Per2	Forward	CACTTGCCTCCGAAATAA
	Reverse	ACTACTGCCTCTGGACTGG
Akt1	Forward	AGAAGAGACGATGGACTTCCG
	Reverse	TCAAACTCGTTCATGGTCACAC
Akt2	Forward	GGCCCCTGACCAGACCTTA
	Reverse	GATAGCCCGCATCCACTCTTC
Atp5pb	Forward	AGTTCCTTTACCCTAAGACTGGT
	Reverse	TTCATGCTCGACTGCTTTACTT
Bnip3	Forward	TGGCAATGGGAGCAGCGTTC
	Reverse	TGGTGTCTGGGAGCGAGGTG

Gene		Primer sequences (5′-3′)
Fabp5	Forward	CGGTCAAAACCGAGAGCACA
	Reverse	TGACGAGGAAGCCCTCATTG
Sirt1	Forward	TGATTG GCACCGATCCTCG
	Reverse	CCACAGCGTCATATCATCCAG
Gapdh	Forward	CTTGTGCAGTGCCAGCC
	Reverse	GCCCAATACGGCCAAATCC

Table S1. Real-Time PCR primer sequences, related to STAR Method.
